# Preliminary Characterization of Extracellular Allelochemicals of the Toxic Marine Dinoflagellate *Alexandrium tamarense* Using a *Rhodomonas salina* Bioassay

**DOI:** 10.3390/md7040497

**Published:** 2009-11-02

**Authors:** Haiyan Ma, Bernd Krock, Urban Tillmann, Allan Cembella

**Affiliations:** Alfred Wegener Institute for Polar and Maine Research, Am Handelshafen 12, 27570 Bremerhaven, Germany; E-Mails: Haiyan.Ma@awi.de (H.M.); Urban.Tillmann@awi.de (U.T.); Allan.Cembella@awi.de (A.C.)

**Keywords:** Alexandrium tamarense, allelochemicals, cell lysis, extracellular compound

## Abstract

Members of the marine dinoflagellate genus *Alexandrium* are known to exude allelochemicals, unrelated to well-known neurotoxins (PSP-toxins, spirolides), with negative effects on other phytoplankton and marine grazers. Physico/chemical characterization of extracellular lytic compounds of *A. tamarense*, quantified by *Rhodomonas salina* bioassay, showed that the lytic activity, and hence presumably the compounds were stable over wide ranges of temperatures and pH and were refractory to bacterial degradation. Two distinct lytic fractions were collected by reversed-phase solid-phase extraction. The more hydrophilic fraction accounted for about 2% of the whole lytic activity of the *A. tamarense* culture supernatant, while the less hydrophilic one accounted for about 98% of activity. Although temporal stability of the compounds is high, substantial losses were evident during purification. Lytic activity was best removed from aqueous phase with chloroform-methanol (3:1). A “pseudo-loss” of lytic activity in undisturbed and low-concentrated samples and high activity of an emulsion between aqueous and *n*-hexane phase after liquid-liquid partition are strong evidence for the presence of amphipathic compounds. Lytic activity in the early fraction of gel permeation chromatography and lack of activity after 5 kD ultrafiltration indicate that the lytic agents form large aggregates or macromolecular complexes.

## 1. Introduction

Phytoplankton in aquatic systems face various survival challenges, some of which result from grazer attack, pathogens and competition with other microalgae. The performance of particular species in these inter-specific interactions is assumed to play an important role in determining which species will survive and even to grow rapidly enough to form aggregations (*i.e.*, “bloom”) at a given time and place. Some algal blooms cause adverse effects including oxygen depletion, reduced water quality, and toxicity, and they are therefore called Harmful Algal Blooms (HABs). Interestingly, many HAB species are regarded as rather poor exploitation competitors in terms of growth rate and/or resource uptake capabilities [1]. In compensation, the hypothesis evolved that a number of HAB species may gain dominance or at least maintain co-existence by production of bioactive compounds, particularly secondary metabolites that affect growth or elicit other physiological responses in other organisms. Such allelochemicals may be targeted to exclude competitors from exploiting limited resources (interference competition), as well as to avoid/reduce predation. In the marine environment, several HAB species from different groups, including cyanobacteria, dinoflagellates, prymnesiophytes and raphidophytes are known to produce such allelopathic chemicals with negative effects, such as inhibition of growth, photosynthesis pathway system II, grazing pressure, and even causing death of other protists [2–4]. In most cases, however, the chemical identity of the compounds remains unknown [5,6].

Marine dinoflagellates in the genus *Alexandrium* are among the most prominent HAB organisms, occurring worldwide, primarily in coastal ecosystems from polar latitudes through temperate waters and including even tropical regimes. *Alexandrium* species frequently form toxic blooms and are well known for their production of biologically active secondary metabolites [*e.g.*, paralytic shellfish poisoning (PSP) toxins, spirolides]. For most of these compounds both the chemical structure and toxicity are well known, mainly because they are toxic to mammals and are therefore of concern to human health [7]. These compounds have been regarded as putative “defensive compounds” because of their high potency in mammalian systems, and they are also suspected to be allelopathic agents responsible for the deleterious effects on metazoan and protistan grazers and algal competitors [8–11]. Whereas the defensive role of the known phycotoxins may remain valid at least in some cases involving metazoan grazers [12], with respect to protistan targets, it is now quite clear that neither PSP toxins nor spirolides are involved in the observed negative effects [13,14]. Thus, it is evident that *Alexandrium* spp., in addition to the neurotoxic PSP toxins, produce still unknown compounds with lytic capacities. These unknown allelochemicals may be significant in explaining the formation and maintenance of *Alexandrium* blooms through direct destructive effects on competing algae or unicellular grazers [13,15–17].

The search for the chemical nature and structure of allelochemical substances that mediate effects among marine protists, based on classical bioassay-driven fractionation have often resulted in loss of activity or led to irreproducible or inconclusive results. The aim of this current study was to investigate the basic properties and the physico-chemical behavior of these poorly examined compounds, tracking activity with an ecologically realistic target bioassay organism. The lytic activity of cell free supernatant of *A. tamarense* stationary cultures was quantified via a microalgal (*Rhodomonas salina*) bioassay system to estimate stability, solubility, extractability, molecular size, and polarity of lytic compounds. Although chemical structures of these allelochemicals have not yet been deduced via these experiments, results of liquid-liquid partition provide evidence for amphipathic compounds, while size-differentiation by ultrafiltration supports the existence of the lytic agents as large aggregates and likely as macromolecular complexes. Preliminary evidence indicates that they are stable over a longer period of time, but nonetheless show unusual unstable characteristics during any further purification, which often result in reduced or no activity.

## 2. Results

### Temporal stability

**Figure 1.**
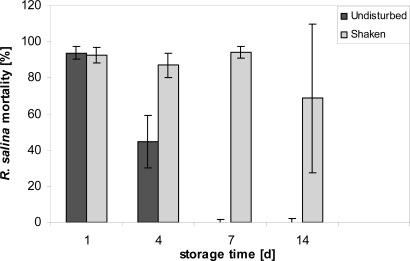
Mortality of *R. salina* [%] exposed to supernatant stored for 1, 4, 7, and 14 days at 15 ºC. Results expressed as triplicate mean ± 1SD.

After storage of diluted (1:10) cell-free culture supernatant in 20 mL glass vials under normal culture conditions (15 °C), lytic activity rapidly declined, with an almost 50% reduction after 4 days and a complete disappearance after 7 days (Figure 1).

**Figure 2.**
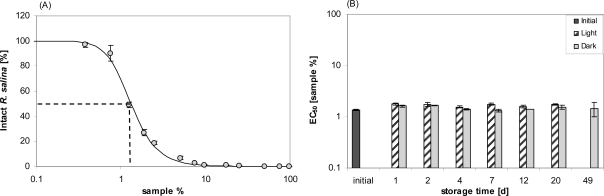
Stability of lytic activity of supernatant after storage under culture conditions. (A) Initial dilution curve of supernatant. Intact *R. salina* (% of control) is plotted against concentration of sample (%, v/v) in the bioassay (log scale). Error bars represent triplicate means ± 1SD. (B) time course of EC_50_ values of samples stored in light (hatched bars) or dark (grey bars). Bars represent duplicate mean ± 1SD.

Lytic activity was however easily reestablished by shaking the containers vigorously before removal of stored supernatant. Even after 14 days the apparent loss of lytic activity could be reestablished by shaking. With a vigorous shaking before sub-sampling, a detailed quantitative experiment on stability under normal culture conditions was then performed. For the initial sample, the dilution curve (Figure 2A) yielded an EC_50_ value of 1.3% (1.2–1.4%; sample concentration in %). EC_50_ values determined after different storage time varied, but with an overall mean of 1.6% did not show any significant decrease in lytic activity over time, neither in flasks kept in the light nor in darkness (Figure 2B). Bacterial concentration in the stored supernatant, as determined for Day 7, was high (light: 1.63 ± 0.35 × 10^7^ mL^−1^: dark: 1.52 ± 0.19 × 10^7^ mL^−1^) and not significantly different between light and dark bottles (*t*-test, n = 2).

### Temperature stability

**Figure 3.**
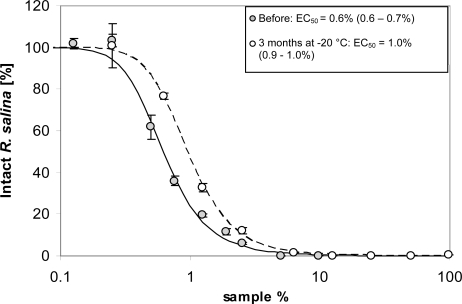
Intact cells of *R. salina* (% of control) exposed to a dilution series of supernatant before (grey dots) and after 3 months storage at −20 °C (white dots). Symbols represent triplicate mean ± 1SD. Figure insert shows corresponding EC_50_ values (95% confidence interval).

As shown above, lytic compounds are stable at room temperature and also did not lose lytic activity upon freezing. After storage at −20 °C for three months, lytic activity was still high (Figure 3) with only minor losses compared to a sub-sample measured before freezing.

Supernatant could also be lyophilized without losing activity; the EC_50_ value of lyophilized and re-dissolved sample was 0.4% (0.4–0.5%), which was only a slight decrease compared to untreated original supernatant [EC_50_ = 0.3% (0.3–0.3%)]. With respect to heat stability, when exposed to higher temperature (95 °C) for 15 min the lytic activity of *A. tamarense* supernatant decreased significantly [EC_50_ = 46.3% (38.8–55.3%)], but only slightly decreased at 60 °C [EC_50_ = 1.5% (1.5–1.6%)] in comparison to room temperature [EC_50_ = 1.1% (1.0–1.3%)]. Lysis of *Rhodomonas* was still visible with samples heated to 95 °C, but 98% of the activity was lost. In undiluted samples, lytic activity was still present when samples were stored for 1 day at 95 °C (*Rhodomonas* mortality: 97 ± 0.1%); after two days at 95 °C, no lytic activity was detected (data not shown; cell numbers of sample not significantly different from control, *t*-test, n = 2).

### pH stability

**Figure 4.**
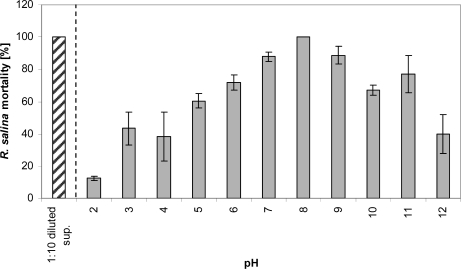
Mortality of *R. salina* cells (%) exposed to supernatant (1:10 diluted) after 3-day storage at pH 2 to 12 at 15 °C. Samples were re-adjusted to pH 8 before performing the bioassay. Results expressed as duplicate mean ± 1SD. sup. = supernatant (positive control).

Lytic activity of a 1:10 diluted supernatant was detected in all samples stored for 3 days at various pH ranging from 2 to 12. Maximum lytic activity was observed at pH 8 and lytic activity decreased with higher and lower pH, but did not disappear completely (Figure 4).

### Solubility

Liquid-liquid partitioning between aqueous supernatant and *n*-hexane revealed formation of a stable emulsion between the aqueous and *n*-hexane phase, which did not disintegrate even after prolonged time. This emulsion layer was therefore also sampled for quantification of lytic activity. Highest lytic activity [EC_50_ = 6.7% (5.3–8.3%)] was indeed found in this layer, whereas no activity was detected in the *n*-hexane phase. Lytic activity in the aqueous phase was intermediate [EC_50_ = 16.8% (9.4–30.0%)] but reproducibility among replicates was rather poor. Generally, lytic activity recovered from liquid-liquid partitioning was only about 10% of the lytic activity of the original supernatant [EC_50_ = 0.5% (0.5–0.5%)]. No lytic activity was detected in any fraction of negative controls from non-lytic strain Alex5 culture supernatant (data not shown).

When the supernatant was pre-acidified to pH 2, and then extracted with *n*-hexane, the mortality of *Rhodomonas* in response to the n-hexane phase was still not different from the control (data not shown). However, the lytic activity in the remaining aqueous phase was also extremely low, and with poor reproducibility, the one-point (3.9 mL) mortality of *Rhodomonas* was 42.0 ± 41.9% (n = 3), at least 10-fold lower than with the original supernatant (at 1:10 dilution yielded mortality of 100 ± 0% (n = 3). Both the *n*-hexane phase and aqueous phase from Alex5 showed no apparent difference from control (data not shown).

**Figure 5.**
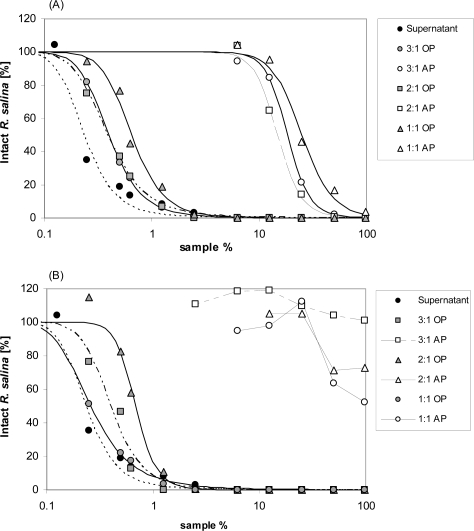
Intact cells of *R. salina* (% of control) exposed to dilution series of supernatant (black dots) or to organic phase (white symbols) and aqueous phase (grey symbols) of supernatant extracted with organic solvents with various composition and ratio. (A) dichloromethane: methanol; (B) chloroform: methanol. AP = aqueous phase; OP = organic phase.

When using extraction solvents with intermediate polarity, both dichloromethane-methanol and chloroform-methanol, did extract components with lytic activity from the aqueous phase. With dichloromethane-methanol mixtures, little but significant activity was still detected in the aqueous phase (Figure 5A). Lytic activity was completely removed from aqueous phase with chloroform-methanol in a 3:1 mixture (Figure 5B). When compared to the original supernatant (EC_50_ = 0.2%) a slight loss of lytic activity was observed for all liquid-liquid partitioning experiments. The lack of detectable lytic activity in negative control samples (K-medium, data not shown) excluded any solvent effects.

### Concentration of lytic compound(s)

**Figure 6.**
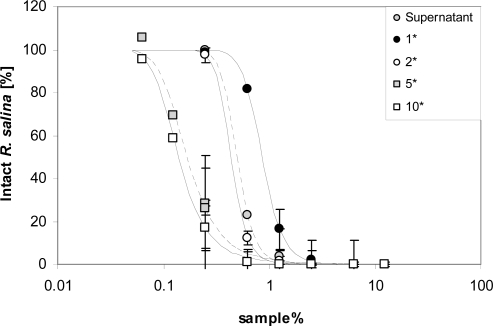
Intact cells of *R. salina* (% of control) exposed to dilution series of supernatant (grey dots) or to chloroform-methanol [3:1 (v/v)] extracted supernatant at 4 concentrations. Results expressed as triplicate mean ± 1SD.

Attempts to concentrate lytic activity by taking up methanolic extracts in less volume than the original supernatant were partially successful. When compared to untreated supernatant, methanol extraction and re-suspension in the same volume yielded > 40% loss of activity (Figure 6). However, lytic activity increased almost linearly in samples re-suspended in a reduced volume, with lytic activity nearly reaching 300% of the untreated supernatant in the five-fold concentrated sample. Further concentration did not to apparently increase lytic activity per unit volume, and reproducibility of the activity of the highest concentrated samples was poor.

### Ultrafiltration

The mass range of the lytic compounds was estimated via ultrafiltration with 5 kDa and 500 kDa cut-off membranes. When small volumes of supernatant (*e.g.*, 20 mL) were initially applied to the ultrafiltration unit with a cutoff 500 kDa membrane, no activity was detected in the filtrate (results not shown).

**Figure 7.**
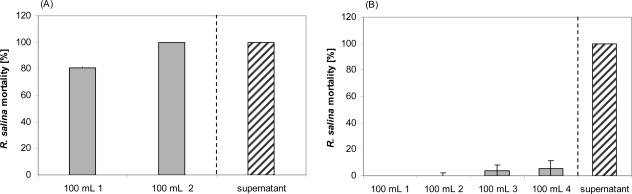
Mortality of *R. salina* exposed to supernatant ultrafiltrate or to supernatant of the same batch (positive control). (A) 500 kDa cutoff filtrate; 100 mL 1 = first 100 mL filtrate of 100 mL supernatant; 100 mL 2 = second 100 mL filtrate of 100 mL supernatant. (B) 5 kDa cutoff filtrate; 100 mL 1 = first 100 mL filtrate of 120 mL supernatant; 100 mL 2 = second 100 mL filtrate of 120 mL supernatant; 100 mL 3 = third 100 mL filtrate of 120 mL supernatant; 100 mL 4 = fourth 100 mL filtrate of 120 mL supernatant. Results expressed as duplicate mean ± 1SD.

**Figure 8.**
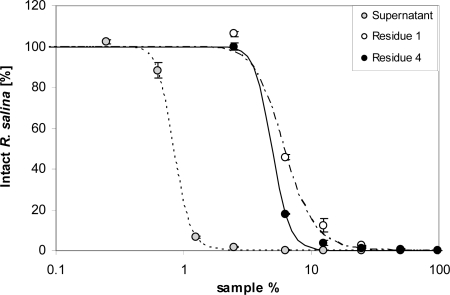
Intact cells of *R. salina* (% of control) exposed to dilution series of supernatant (grey dots) or residues from 5 kDa ultrafiltration from the first and fourth run of filtration (see text). Results expressed as duplicate mean ± 1SD.

However, when 100 mL supernatant were passed through a 500 kDa membrane, activity was found in the initial filtrate and increased in filtrates of subsequent runs (Figure 7A). Ultrafiltration through a 5 kDa cut-off membrane showed no significant activity in any of the four filtrates, even though four x 100 mL of supernatant were subsequently filtered (Figure 7B). Lytic activity in residues after 5 kDa ultrafiltration was considerably lower compared to untreated supernatant (Figure 8). A drastic decrease was observed between supernatant (EC_50_ = 0.8%) and the residue of the first run (EC_50_ = 6.1%), whereas subsequent ultrafiltration runs did not yield a further decrease of lytic activity (EC_50_ = 4.9%).

### Gel permeation chromatography (GPC)

**Figure 9.**
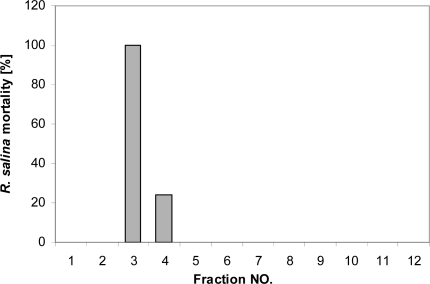
Mortality of *R. salina* (%) exposed to fractions collected after gel permeation chromatography. The first two fractions correspond to the column void volume.

When 5 mL of concentrated supernatant (extracted from 50 mL supernatant by chloroform-methanol (3:1) and concentrated to 5 mL) were applied to a Superdex 30 gel permeation column for size determination, lytic activity was primarily found in Fraction 3 and to a lesser extent in Fraction 4 (Figure 9). These were the first fractions collected after the void volume. Quantitative evaluation of the GPC method revealed very poor recovery of lytic activity. An equivalent to 150 mL supernatant (extracted and concentrated to 15 mL) was injected in 3 subsequent runs and pooling of the corresponding fractions allowed for a detailed quantification of lytic activity by preparing dilution curves. Corresponding EC_50_ values [Supernatant = 2.2% (2.0–2.5%); GPC fraction = 7.3% (6.4–8.3%)] indicate that only 1% of the activity was recovered after GPC.

### Clean-up by solid phase extraction (SPE)

Initial clean-up experiments on the cell-free culture supernatant by reversed phase SPE C-18 cartridge yielded no apparent mortality of *Rhodomonas* after treatment with 100% methanol SPE eluent of sample loading volumes up to 100 mL.

**Figure 10.**
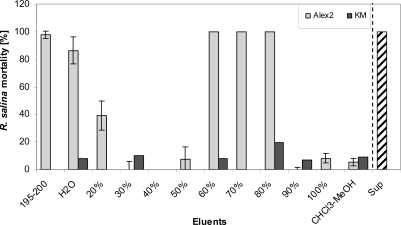
Mortality of *R. salina* (%) exposed to different eluate fractions obtained after reversed phase solid phase extraction (SPE) of 200 mL supernatant (Alex2, grey bar) or 200 mL K-medium (negative control, KM (K-medium), black bar): 195–200 = last 5 mL sample load eluate; H_2_O = water wash; 20–100% = percentage of aqueous methanol; CHCl_3_-MeOH = 1:1 (v/v) mixture of chloroform/methanol; Sup = untreated supernatant (positive control). Results expressed as triplicate mean ± 1SD.

Yet 100% mortality of *Rhodomonas* was observed when at least 200 mL of supernatant were loaded onto the SPE cartridge. Sequential elution of the SPE cartridge with eluents of increasing (10% increments) methanol content revealed two distinct fractions containing lytic activity. An initial hydrophilic fraction contained lytic activity spread over the five mL (195 to 200 mL) of the sample load eluate, the water wash and the 20% methanol fraction. Fractions containing 30 to 50% methanol did not show any lytic activity. A second more lipophilic fraction with lytic activity was detected in the eluates with 60% to 80% methanol (Figure 10).

**Figure 11.**
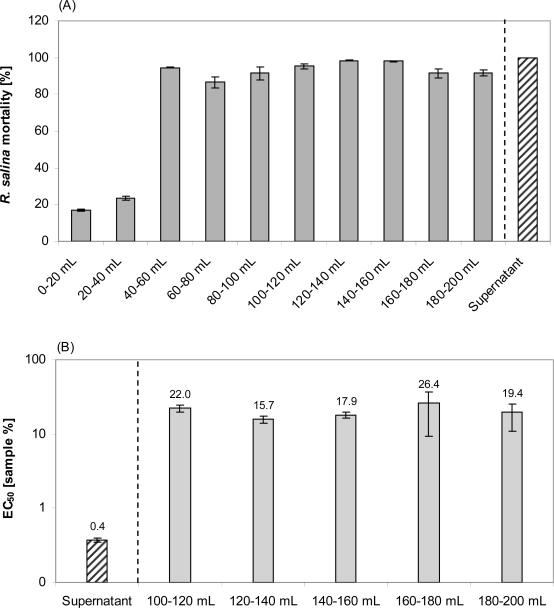
*R. salina* exposed to consecutive eluate fractions (as indicated below the bars) of reversed phase solid phase extraction (SPE) of 200 mL supernatant or exposed to untreated supernatant (positive control). (A). *R. salina* mortality from one-point bioassay. (B). EC_50_ and 95% confidence interval. Results expressed as duplicate mean ± 1SD.

**Figure 12.**
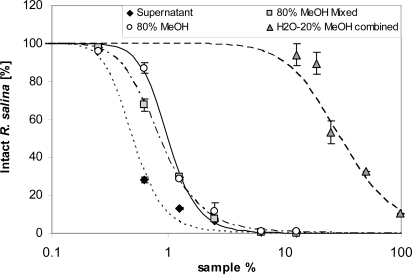
Intact cells of *R. salina* (% of control) exposed to different eluate fractions of 200 mL supernatant obtained with reversed phase solid phase extraction (SPE) or to dilutions of untreated supernatant (black dots, positive control): 80% MeOH = 5mL aliquot of 80% methanol fraction, evaporated and dissolved in 5 mL K-medium; 80% MeOH mix = 5 mL aliquot of 80% methanol fraction evaporated and dissolved in 5mL of last 20 mL sample load eluate; H_2_O-20% MeOH combined = 10 mL water and 10 mL 20% methanol fraction evaporated and dissolved in 20 mL K-medium. Results expressed as duplicate mean ± 1SD.

Lytic activity appeared in the sample load eluate only after 40 mL supernatant was applied (Figure 11). Interestingly, the EC_50_ of the sample load eluate, separately measured for consecutively collected fractions (20 mL each), did not change (Figure 11). With an average EC_50_ of 20.3%, lytic activity in the sample load eluate accounted for about 2% of the total lytic potency of the supernatant. The 80% methanol Fraction 2 was much more lytic (EC_50_ = 1.0%) than the water-20% methanol Fraction (EC_50_: 29.9%) (Figure 12). When taking the different volumes into account, the lytic activity of Fraction 2 was about 2.5% of the total lytic activity of 200 mL supernatant (EC_50_ = 0.5%) applied to the SPE cartridge. Total loss of activity after SPE was calculated as 96%. Re-dissolving Fraction 2 in the sample load eluate did not significantly increase lytic activity, indicating that there is no major synergistic effect of lytic compounds of the separate fractions. Eluates from SPE of supernatant of a culture of the non-lytic clone Alex5 (negative control) yielded no lytic activity (not shown), therefore sample treatment artifacts causing cell lysis of *Rhodomonas* can be excluded.

### Effects of SPE fraction storage conditions on lytic activity

**Figure 13.**
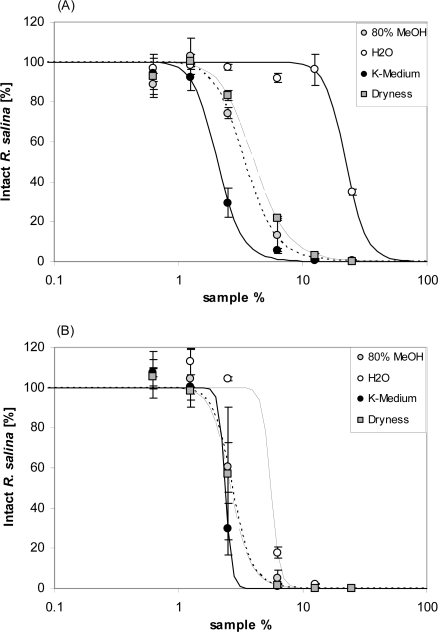
Intact cells of *R. salina* (% of control) exposed to four 80% methanol SPE fractions of supernatant, each stored at different conditions (for explanation see text): 80% MeOH = unchanged; H_2_O = solvent evaporated and re-dissolved in deionized water; K-Medium = solvent evaporated and re-dissolved in K-medium; Dryness = solvent evaporated. (A) after 2 days storage. (B) after additional 2 days storage in K-medium. Results expressed as duplicate mean ± 1SD.

Storage of lytic active eluates from reversed phase SPE in seawater-base culture medium yielded markedly different effects, depending upon the specific storage conditions. Re-suspension of dried SPE fraction in seawater based K-medium resulted in higher lytic activity (lowest EC_50_ value, Table 1) than for fractions kept for two days as dry residue or in 80% aqueous methanol. In contrast, storage of SPE fractions in deionized water drastically decreased lytic activity in the subsequent bioassay to such an extent that EC_50_ was almost one tenth of the lytic activity of fractions stored in K-medium (Figure 13A, and Table 1). However, after additional 2 days stored in seawater culture medium, lytic activity of this sample was largely restored (Figure 13B and Table 1). A comparable but smaller increase in lytic activity upon additional storage in seawater (significant for the treatment “dryness”) was observed for the two other samples.

### Reversed phase high performance liquid chromatography (HPLC)

Reversed phase chromatography of SPE pre-cleaned supernatant (80% methanol eluate) on a lipophilic C8 phase yielded an active fraction between 18 and 19 min. None of the other fractions collected in one minute increments showed any lytic activity in the *Rhodomonas* bioassay. The 18 to 19 min window of the chromatogram also revealed a peak in the diode array detector (DAD). The UV spectrum of this peak had an absorption maximum at 236 nm. However, the same UV spectra were also observed in neighboring peaks, which showed no lytic activity of the lytic Alex2 supernatant, nor of the same chromatographic region of the non-lytic Alex5 supernatant.

### Dry mass equivalent

The EC_50_ was reached with 1.9 mg (1.7–2.1 mg) of dried supernatant. After clean-up by reversed phase SPE, the EC_50_ was equivalent to 78 μg (60–101 μg) of the 80% methanol fraction. The same activity was achieved with 31 μg (28–34 μg) of the lytic HPLC fraction (18–19 min). These results indicated that 96% of interfering substances in the supernatant were removed by the SPE clean-up. Salt removal from the supernatant mainly contributes to this reduction of dry mass equivalent. Further separation by C8 HPLC additionally excluded approximately another 60% of dry mass not related to lytic activity.

## 3. Discussion

Species of the genus *Alexandrium* are well known for their production of biologically active secondary metabolites. For some of these compounds, particularly the neurotoxins associated with paralytic shellfish poisoning (PSP) (saxitoxin and derivatives) and the macrocyclic imine toxins spirolides, both the chemical structures and toxicity are well known, mainly because these compounds are toxic to mammals. In contrast, other compounds are well described in terms of their bioactivity toward ecologically meaningful targets (*e.g.*, lytic activity against accompanying protists), but detailed structural information is completely lacking. Whereas the known phycotoxins (PSP, spirolides) are mainly stored inside the cells, the toxicity of *Alexandrium* to other protists is caused by lytic compounds excreted to the medium, facilitating direct contact to competing and interacting protistan species. Consequently, in our attempts to characterize allelochemicals of *A. tamarense* with lytic effects on marine protists, we exclusively focused on extracellular compounds to be found in the culture medium. This approach is justified *per se* by the term “allelopathy”, coined by Molisch [18] as defining effects through the production of chemicals that are released into the environment to affect other species, and by the notion that compounds enclosed inside cells cannot directly affect co-existing planktonic species in the absence of cell lysis (death). The high ecological relevance of investigating potential allelochemicals in the extracellular matrix, however, is saddled with all the disadvantages associated with extracting diluted metabolites from aqueous media such as seawater. In order to gain a maximum concentration of lytic compounds in the cell-free fraction, we sampled dense, stationary cultures, which have been shown to contain highest lytic activity (unpublished results). Centrifugation to separate cells and cell-free supernatant was preferred because filtration was shown to drastically reduce lytic activity [4], probably both due to filter pore-size restriction and adsorption to filter material.

The successful search for allelochemicals is also highly dependent upon the appropriate choice of bioassay to follow biological activity. Rather than the commonly used brine shrimp or mammalian cell lines, we selected the small cryptophyte *Rhodomonas salina*, which co-occurs with *Alexandrium* in coastal marine systems and thus is an ecologically relevant bioassay organism. Furthermore, previous experiments [14,39] have shown that *R. salina* is rapidly susceptible to easily quantifiable allelochemical interactions with other protists and can therefore serve to follow bioactivity for physico-chemical characterization.

Previous attempts to purify and isolate these allelochemical compounds by classical bioassay-driven fractionation often resulted in loss of activity and erratic and irreproducible results. For this reason we decided to carefully characterize the properties and behavior of these compounds before starting their isolation. As a prerequisite for effective bioassay-guided fractionation it is necessary to obtain basic information on physico-chemical properties of the target compounds. The stability of the compounds, or more precisely the stability of the biological effect quantified with the bioassay is important. For example, toxic compounds of the haptophyte *Prymnesium parvum* have been shown to rapidly degrade with time, especially under the influence of light [19,20]. Lability of compounds is likely to have also exacerbated identification of potential toxins produced by the putatively toxic dinoflagellate *Pfiesteria piscicida* [21]. In contrast, the high stability of extracellular lytic compounds from *A. tamarense* under moderate conditions (*e.g.*, normal room temperature and ambient light) significantly relaxes requirements for further treatment for purification and identification. Heat stability of lytic activity was also high; almost no loss of activity after heating at 60 °C indicates that enzymatic activity is not involved in lysing target cells. Moreover, temporal stability in the presence of bacteria (Figure 2B, Day 7) showed that lytic compounds are refractory to bacterial degradation (at least of the bacterial consortium typically present in the culture medium). From an ecological point of view, this stability is important because it increases the probability that biologically active threshold concentrations of these compounds in the water are reached and maintained for the benefit of the producing organisms.

Perhaps surprisingly given the complexity of the probable macromolecular structures lyophilization of supernatant hardly altered lytic activity after resuspension in culture medium. Yet the lytic effect of cell-free supernatant appeared to rapidly decrease (Figure 1) when samples were collected with minimal physical disturbance, whereas after vigorous shaking lytic activity was fully recovered. This finding may accord with an observed “loss” of hemolytic activity of putative toxins of the haptophyte *Prymnesium parvum*, depending on the surface/volume ratio of different storage containers [22], which was explained by a movement of monomers of these amphipathic molecules to the surface.

Amphipathic compounds, such as detergents and surfactants, are generally large molecules composed of an ionic or non-ionic polar head and a hydrophobic part, which promote the formation of aggregates (micelles, bilayers, monolayers, hexagonal or cubic phase) in aqueous media. Most important, physico-chemical behavior and properties of amphipathic compounds are strongly modulated by self-association or by their binding to other lipophilic aggregates. Monomers and aggregates can be interconverted as a function of pH, temperature, ionic strength, and concentration of the compounds (for a review, [23]), the latter being described by the critical micelle concentration below which compounds mostly occur as monomers.

Bioactive compounds from other toxic marine flagellates, including species of *Prymnesium*, *Amphidinium* and *Karlodinium*, have been shown to behave like amphipathic molecules [22,24,25], which also seems to be the case for the lytic compounds of *Alexandrium*. In addition to the observed loss of activity after filtration [4] and the “pseudo-loss” of undisturbed and low-concentrated samples, the observed high lytic activity of the emulsion between aqueous and *n*-hexane phase after liquid-liquid partition is a strong indication for amphipathic compounds. Likewise, attempts to estimate molecular size are largely hampered for micelle-forming compounds. The loss of activity after filtration using small pore-sized filters, appearance of lytic activity in the first fraction of gel permeation chromatography and lack of activity after 5 kD ultrafiltration are indications that the lytic agents are unusually large complexes because of micelle formation.

Fatty acids have been reported to be associated with hemolytic, antibacterial or anti-algal effects in several dinoflagellate species [26–29]. However, *n*-hexane extraction of acidified supernatant did not show any lytic activity in the bioassay, from which we conclude that fatty acids alone are not responsible for *Alexandrium* extracellular lytic activity.

The results further show that, although temporal stability of the compounds is high, substantial losses seem to be unavoidable during purification. This may be generally explained by a “stickiness” of the compounds to various surfaces, as has been described for filter material [4] and differentially for plastic containers compared to glass (unpublished data). A high adsorption of bioactive compounds to various materials has also been observed for karlotoxins [30] and might be explained by high charge of the micelle surface, yet another indication of the amphipathic nature of these compounds.

It is also interesting to consider how storage conditions affected lytic activity. Pretreatment of the partly purified lytic fraction in seawater – compared to ion-free water or different solvents - increased the activity (Figure 13). A similar phenomenon is described for *Prymnesium* toxins. When Prymnesium toxin B was diluted in methanol, ethanol or chloroform-methanol (2:1, v/v), or distilled water, the resultant hemolytic activity was approximately 10-fold decreased when compared to the sample dissolved in 0.85% NaCl [22]. Ions thus are able to modify lytic activity of amphipathic compounds, probably by affecting the solubility of relatively non-polar compounds in water or their partitioning between water and organic solvents [31].

Apparently, two distinct lytic fractions with different polarity (high polarity: eluted with 20% methanol; low polarity: eluted with 80% methanol) were separated via reverse SPE (Figure 10). EC_50_ values of both fractions indicate that lytic activity of the less polar fraction was about 15 fold higher. The fact that some lytic activity already appears very early in the sample load eluate (Figure 11) is a strong indication that this fraction is very hydrophilic and hardly retained on the lipophilic material of the C-18 SPE cartridge. This hypothesis is supported by the observation that the amount of lytic activity in the waste did not change after an initial increase (no mortality in the first 40 mL, and then 100% mortality thereafter) and accounts for only a minor part (*ca*. 2%) of total activity of the supernatant. In contrast the less polar fraction could only be detected after loading more than 100 mL sample to the C-18 SPE cartridge suggesting a very strong absorption of this fraction to the cartridge material.

Nevertheless, more than 95% of total activity of the supernatant applied to SPE could not be eluted from the column. It is unlikely that this loss was caused by nullifying synergisms of two different compounds now separated into two different fractions, as simple mixing of the two fractions did not significantly increase the lytic activity. A strong adsorption of the non-polar fraction to C-18 material, as has been found for all kind of surfaces, might be responsible for the large loss.

The fact that lytic activity elutes in a discrete retention window between 18 and 19 minutes from a reversed C8 phase but does not show any specific absorption of UV radiation, may be explained by two alternative hypotheses: 1) lytic compounds are active at very low concentrations and coelute with compounds unrelated to lytic activity on this chromatographic system, or 2) lytic compounds belong to a suite of chemically similar (but non-lytic) compounds and only small modifications, which do not result in altered UV absorption, are responsible for lytic activity. Both hypotheses will have to be tested in future work.

A still unresolved problem is the loss of activity after purification and separation of supernatant. Similar effects have been described for karlotoxins produced by *Karlodinium* spp. and for postulated exotoxins produced by *Pfiesteria piscicida.* Whereas karlotoxins have a high affinity to organic polymers, to such an extent that they can be extracted from aqueous media by filtration over polymer filters and eluted with methanol [30], the proposed *Pfiesteria* toxins consist of a metal complex and toxicity seems to be caused by production of free radicals and redox reactions [21]. Both characteristics, even though similar to karlotoxins and the bioactive components of *Pfiesteria*, can be excluded for lytic compounds from *Alexandrium*. The loss of activity after contact with organic polymeric material like SPE cartridges or filters is irreversible (in contrast to karlotoxin). On the other hand there is some parallel to the bioactive components of *Pfiesteria* because of the evidence that metals may play a role in *Alexandrium* lytic activity. This is supported by the fact that lytic fractions incubated in seawater-base culture medium show higher activity than fractions incubated in deionized water prior to the bioassay. Unlike for *Pfiesteria*, *Alexandrium* lytic activity is very stable and does not degrade easily, indicating that the bioactive agents of *Pfiesteria* and *Alexandrium* and their physico-chemical properties are completely different.

There are three other recent studies dealing with exotoxins of the genus *Alexandrium*. A heat-labile exotoxin which showed potent toxic effect on brine shrimp (*Artemia salina*) has been reported from *A. minutum* [32], and a hemolytic exotoxin of *A. taylori* has been described as proteinaceous, with a molecular weight of more than 10 kDa [33]. A novel high molecular weight (about 1,000 kDa) exotoxin (both hemolytic and cytotoxic) was reported from *A. tamarense* as most likely a polysaccharide-based compound [34]. The molecular weight estimated by Yamasaki *et al*. [34], however, could not be confirmed by our study, as lytic activity could be detected in the 500 KDa cut-off membrane filtrate. This might be due to the difficulties to correctly estimate molecular size of amphipathic compounds, or to the presence of different compounds in different species and/or strains of *Alexandrium*. Both Emura *et al.* [33] and Yamasaki *et al.* [34] used hemolytic and cytotoxicity assays in the search for novel ichthyotoxic compounds produced by blooms of *Alexandrium* species and affecting fish stocks in aquaculture. These studies did not specifically address or test for bioactive compounds associated with allelochemical interactions among protists. It is therefore not unlikely that the compounds represented by these previous studies have different properties and are unrelated to the lytic compounds we tested for in our *Rhodomonas* bioassay.

## 4. Experimental Section

### Cell culture

One clonal isolate of *Alexandrium tamarense* (Alex2) was selected as the source of lytic compounds from a larger collection of >60 clones of the North American ribotype (Group 1, [35]) of this species isolated simultaneously in May, 2004 from the Scottish east coast of the North Sea (56° 05′ 47″ N and 1° 42′ 35″ W). The clones were isolated by micro-capillary pipette from a single vertical net haul (20 μm mesh) from 20 m depth [36] and maintained as unialgal stock cultures under standardized growth conditions. Dinoflagellate cultures were grown in K-medium [36,37], supplemented with selenite [38], prepared from sterile-filtered (VacuCap 0.2 μm Pall Life Sciences) natural North Sea seawater (salinity 32 psu) in 1,000 mL Erlenmeyer flasks. Cultures were maintained under controlled conditions at 15 °C under cool-white fluorescent light at a photon flux density (PFD) of 100 μmol photons m^−2^ s^−1^ on a 16 h light: 8 h dark photocycle. Alex2 was selected based on its high lytic activity as comparatively quantified in previous experiments [36,39]. In some experiments, another isolate of this collection (Alex5), which does not produce lytic compounds in measurable amounts [39], was used as a negative control.

### Sample harvest and preparation

Different techniques for maximum yield of extracellular lytic activity from *A. tamarense* cultures, including the optimal harvest time for extracellular lytic compounds, were previously compared (unpublished data). Based on these results, samples were prepared by separating cells from the culture medium by centrifugation (50 mL Sarstedt tubes, Eppendorf centrifuge 5810R, 3,220 × *g*, 15 °C for 10 min) of dense stationary phase cultures (ca. 20 x 10^3^ cells mL^−1^). Supernatant of several tubes was combined in glass vessels and either used directly or stored in the fridge (4 °C for weeks).

### Chemical reagents

Water was deionized and purified (Milli-Q, Millipore GmbH, Eschborn, Germany) to 18 MV cm^−1^ quality or better. Methanol (LiChrosolve for HPLC, and p.a. for all other experiment), chloroform (p.a.), dichloromethane (p.a.), hydrochloric acid (HCl, 32%, p.a.) and *n*-hexane (p.a.) were purchased from Merck (Darmstadt, Germany), and sodium hydroxide (NaOH, p.a.) were from Sigma (Aldrich, Germany).

### Bioassay system

A microalgal bioassay system was used to quantify lytic activity based on the response of the photosynthetic cryptophyte *Rhodomonas salina* (Kalmar culture collection strain KAC 30, hereafter named “*Rhodomonas*”) as the target species. *Rhodomonas* was cultured under the same conditions as described for Alex2. In this bioassay, intact *Rhodomonas* cells were microscopically counted after short-term treatment with one or several dilutions of a sample and compared to cell numbers of a control (treated with culture medium). The bioassay was set up in a total volume of 4 mL in 6 mL glass vials. Each sample was spiked with 0.1 mL of a *Rhodomonas* culture which was adjusted (based on microscope cell counts) to 4 × 10^5^ cells mL^−1^ to a final start concentration of 1 × 10^4^ mL^−1^ of the target cells in the bioassay. Samples were then incubated for 3 h in the dark at 15 °C. Subsequently, samples were fixed with 2% Lugol’s iodine solution and concentration of intact target cells was determined. All counts were performed with an inverted microscope (Zeiss Axiovert 40C, Göttingen, Germany) in 2 mL counting chambers. The volume set up for cell counts was 0.5 mL. A sub-area of the chamber corresponding to at least 600 *Rhodomonas* cells in the control was counted. In order to quantify lytic effects, only intact cells of the target species were scored.

The bioassay was performed in two different ways. For the “one-point” approach to even detect a minor decline of lytic activity, one concentration of sample was applied and percent mortality (by comparing cell number to a control) were calculated as a measure of lytic activity of the sample, and in these experiments, the supernatant was properly diluted before the experiment to have a start activity of the compounds close to 100% target mortality. For detailed quantification, a dose response curve was determined, which allows for a calculation of EC_50_ concentration, *i.e.*, the concentration of sample (as %, v/v) affecting 50% of the targets. As performing dose-response curves is rather time consuming, simple one-point measurements of target mortality was used in some of the following experiments.

In one-point bioassays, mortality of *Rhodomonas* was calculated as:

$$\text{mortality}\;=\;[1-({\text{Rho}}_{\text{final}}/{\text{Rho}}_{\text{control}})]*\;100\%,$$

where Rho_final_ is the experimental final *Rhodomonas* cell concentration, and Rho_control_ the final target cell concentration in controls.

When performing dilution series experiments, the percentage of intact *Rhodomonas* followed a sigmoidal decreasing pattern when plotted against logarithmic-scaled sample concentration (as %, v/v)). Estimates of EC_50_, *i.e.*, the volume of Alex2 supernatant yielding a 50% decline in target cell concentration, were determined by fitting the data points to the following equation using the non-linear fit procedure of Statistica1 (Statsoft, Hamburg, Germany) for Windows:

$${\text{N}}_{\text{final}}\;=\;{\text{N}}_{\text{control}}/[1\;+\;{\left(\text{X}/{\text{EC}}_{50}\right)}^{\text{h}}]$$

where N_final_ is the experimental final target percent intact cells (0–100%), N_control_ the final target cell percentage in controls (100%), X the logarithmic-transformed percentage of volume of Alex2 supernatant in the 4 mL test system, and EC_50_ and h are fit-parameters. Results are expressed as EC_50_ (sample %) including 95% confidence intervals.

For one-point measurements, *t*-test statistics with a significance level of 0.05 were used to identify significant differences between number of intact cells in samples and control, and also to compare replicate EC_50_ values. When single dilution curves were available, comparison of EC_50_ values was based on their 95% confidence intervals.

### Temporal stability

Stability of lytic activity under normal culture incubation conditions was tested by filling 24 glass vials (Wheaton, 20 mL) each with 15 mL of 1:10 diluted (with K-medium) supernatant and following the decline in activity over time with a one-point bioassay. Incubated samples were stored in a culture room at 15 °C for 1, 4, 7 and 14 days, respectively. After each respective incubation period, six vials were removed; 3.9 mL sub-samples were taken without perturbation from three vials, whereas the other subset of three vials was shaken vigorously before sub-sampling. A *Rhodomonas* bioassay of these sub-samples was performed as described above.

A fully quantitative estimation of stability under normal culture conditions was then performed as follows: 500 mL of supernatant was prepared and apportioned into 5 × 100 mL in 100 mL Duran glass flasks. From one flask, lytic activity was quantified from an 11 points dilution curve (each dilution in triplicate). The other flasks (in duplicates) were stored under normal dinoflagellate culture conditions (as described herein). One of the two sets of flasks was kept dark by wrapping with aluminum foil. After 1, 2, 4, 7, 12, 20 or 49 days, sub-samples of each flask were taken after vigorously shaking the flasks to perform a bioassay and the activity was determined from an 11-point dilution curve (each dilution in duplicate). For the final sampling (after 49 days), samples from flasks held in the light had to be omitted because a low but substantial number of cells was present, which had obviously originated from a few cells still present in the culture supernatant. After 7 days, 1 mL sub-samples from each flask were fixed with formalin (2% final concentration) for determining bacterial abundance according to a standard technique with acridine orange staining [40]. Stained bacteria were counted under an epifluorescence microscope (Zeiss Axiovert 2 Plus, Göttingen, Germany) with a 100× magnification oil immersion objective. At least 400 cells per filter (except for blank filters) were counted.

### Temperature stability

Effects of freezing were tested by a quantitative comparison of lytic activity of supernatant before and after prolonged (3 month) storage at −20 °C in the dark. A total of 200 mL supernatant was prepared and a standard bioassay to quantify lytic activity was performed (in triplicate) with a series of 9 dilutions. Subsequently, an aliquot of 50 mL of the remaining supernatant was stored in the freezer at −20 °C in a glass bottle. After 3 months storage, the sample was thawed at room temperature and lytic activity was quantified from a 9-point dilution curve (in triplicate).

Frozen supernatant (90 mL at −20 °C) was lyophilized by a Freeze Dryer Beta I (Martin Christ, Osterode am Harz, Germany) at −20 °C and ca. 0.004 mbar vacuum. After three days, the lyophilized sample was re-dissolved in 90 mL water, and lytic activity was quantified by an 8-point dilution series bioassay and compared to untreated supernatant, which was stored at 4 °C during lyophilization.

Short-term heat stability was tested by incubating 20 mL supernatant in 20 mL glass vials in duplicate at room temperature (ca. 20 °C) as control, and at 60 and 95 °C, each in a water bath for 15 min, respectively. The heated samples were rapidly cooled with ice to room temperature. From each sample, a *Rhodomonas* bioassay was performed as described above for the 9-point dilutions, with each dilution set up in triplicate. Based on the results of the first experiment, a second experiment was performed to test the long-term heat stability at 95 °C. Fifteen mL supernatant of another batch was incubated in duplicate in 20 mL glass vials in an oven at 95 °C for 1 and 2 days, respectively. After cooling the sample to room temperature, lytic activity was estimated with a one-point measurement of undiluted sample as described above.

### pH stability

The effect of storage at different pH on extracellular lytic compounds was tested as follows. Fifteen mL of 1:10 diluted supernatant in duplicate were stored in 20 mL glass vials at pH ranging from 2 to 12. The pH was adjusted with NaOH (4 M and 0.1 M) and HCl (32% and 3.2%). Samples were stored for 3 days in the dark at 15 °C, and then adjusted with NaOH and HCl to pH 8. The increase in volume due to this adjustment was always below 1 mL. The *Rhodomonas* bioassay was carried out by applying 2 mL sub-samples within the 4 mL bioassay system.

### Solubility

Partitioning of lytic activity after solvent extraction was analyzed as a first step towards a chemical characterization. The prime task was to exclude the possible negative effect of *n*-hexane. 10 mL supernatant from both Alex2 and Alex5 cultures harvested in stationary phase (ca. 20 × 10^3^ cells mL^−1^) was extracted twice with 5 mL *n*-hexane (in triplicate); aqueous and *n*-hexane phases were separated by centrifugation (Eppendorf, 3220 × g, 5 min, 15 °C), and then evaporated by rotary evaporation at 40 °C (Rotavapor, Büchi, Flawil, Switzerland) to dryness. The aqueous phase residues were redissolved in 10 mL water, whereas *n*-hexane phase residues were brought up in 10 mL K-medium. A one-point (3.9 mL) bioassay of each sample was performed. Accordingly, 5 mL supernatant in a 50 mL plastic Sarstedt tube was mixed with the same volume of *n*-hexane in triplicate, then the samples were centrifuged (3,220 × g, 5 min, 15 °C). Four mL of the *n*-hexane phase (top) and of the aqueous phase (bottom) were removed, and the 2 mL emulsion between the layers was also separately removed. All three phases were dried by rotary evaporation and *n*-hexane extracts and aqueous residues were dissolved in 4 mL K-medium or water, and the emulsion was taken up in 1 mL water and 4 mL K-medium, respectively. Bioassays were performed with a 6-point dilution series after sample storage at 4 °C for 2 days.

Fifteen mL Alex2 as well as Alex5 supernatant in triplicates were acidified with 1 and 0.1 N HCl to pH 2.0. Then the samples were extracted three times with 5 mL *n*-hexane. The *n*-hexane phase was removed without interrupting the inter-phase, and then dried by rotary evaporation, and finally dissolved in 15 mL K-medium. The remaining aqueous phase was dried by rotary evaporation, and dissolved in 15 mL water, and finally adjusted to pH 8.0 with 1 and 0.1 N NaOH. One-point bioassays (3.9 mL) were performed with samples from both *n*-hexane and aqueous phases, with original Alex2 supernatant as positive control.

Chloroform, dichloromethane, methanol and mixtures of these solvents, which are less hydrophobic, were tested in different ratios (chloroform or dichloromethane: methanol = 3:1; 2:1; 1:1 (v/v)). Twenty mL supernatant were mixed with 5 mL of each solvent combination, and subsequently centrifuged (3,220 × g, 5 min, 15 °C). Organic phases were carefully removed from the bottom with a pipette. The aqueous phase was extracted twice more with 5 mL each of the respective organic solvent, and then dried by rotary evaporation and dissolved in 20 mL water. The organic phases were combined and dried by rotary evaporation and finally dissolved in 20 mL K-medium. Lytic activity of both, organic and aqueous phases was measured by *Rhodomonas* bioassay with a 10-point dilution series. Similarly, to exclude the negative effect of the organic solvents, the same procedure was performed with the culture medium as negative control, and a one-point (3.9 mL) bioassay was performed.

### Concentration of lytic compound(s)

To concentrate lytic activity for further analysis, 30 mL supernatant were extracted three times with 5 mL chloroform/methanol (3:1 (v/v)) in triplicate. After evaporation to dryness, the residue of the organic phase was taken up in 3, 6, 15, 30 mL K-medium, respectively, to obtain a calculated 10, 5, 2, and 1-fold concentrated supernatant. Lytic activity was quantified with a 7-point dilution series.

### Ultrafiltration

Ultrafiltration was performed with an ULTRAN^®^-MiniFlex cross flow system (Schleicher & Schuell MicroScience, Dassel, Germany) with a filter area of 24 cm^2^ per cassette, operated at a pressure between 1.0 to 1.8 bar. Ultrafiltration membranes PES 500 (cut-off 500 kD) and PES 5 (cut-off 5 kDa) (Schleicher & Schuell MicroScience, Dassel, Germany) were used. For the 500 kDa membrane, 200 mL supernatant were applied until the retentate reservoir was empty. The filtrate was collected in two separate aliquots of 100 mL. Lytic activity of these two filtrate fractions was tested by taking 3.9 mL sub-samples (in duplicate) for a one-point *Rhodomonas* bioassay. With the 5 kDa cut-off membrane, 120 mL Alex2 supernatant were applied to the system, and 100 mL filtrate was collected. The residue of 20 mL was subsequently made up with 100 mL K-medium. This procedure was repeated three times. Sub-samples of 3.9 mL each were taken to estimate lytic activity in all four filtrates. A sub-sample of the first and fourth residue, as well as the supernatant batch applied to the PES 5 system, was tested with the *Rhodomonas* bioassay in a 9-point dilution series.

### Gel permeation chromatography (GPC)

Fifty mL of supernatant were extracted with chloroform/methanol [3:1 (v/v)] as described above, and the residue of the rotary-evaporated organic phase was dissolved in 5 mL water. The concentrated sample was applied to a Superdex 30 GPC column (Hiload 16/60, 13,000 plates), with 40% methanol as eluent. Each run was performed for 120 min at a flow of 1.15 mL per min. For each run, 12 fractions were collected (10 min for each tube), and rotary-evaporated to dryness. The residues were dissolved in 5 mL K-medium and incubated for two days. A one-point (3.9 mL) bioassay was performed as described above to determine lytic activity in each fraction, together with supernatant of the same batch as positive control. Subsequently, 150 mL supernatant was concentrated the same way to 15 mL, and was applied to the GPC column by injection in three subsequent portions. Corresponding fractions from all three runs were pooled and rotary-evaporated to dryness, and finally the lytic activity as well as supernatant of the same batch was tested via bioassay with a 7-point dilution series.

### Clean-up by solid phase extraction (SPE)

Lytic compounds were extracted from the supernatant by reversed-phase SPE cartridge (LC-18, 500 mg/6mL, Sigma-Aldrich, Deisenhofen, Germany). The cartridges were conditioned by 15 mL chloroform/methanol (3:1 (v/v)) and 15 mL 100% methanol and equilibrated with 15 mL water. First, in order to define a minimum volume of supernatant to be applied to SPE, a series of four volumes (10, 50, 100, and 200 mL) of supernatant were applied to SPE cartridges and each eluted with 10 mL water, 40% methanol, 100% methanol, and chloroform/methanol (3:1 (v/v)). Based on the preliminary test, 100% methanol fractions were targeted, and dried by rotary-evaporation, re-dissolved in 5 mL K-medium and tested as a one-point bioassay of undiluted sample for lytic activity. In the next experiments, 200 mL supernatant in triplicate as well as 200 mL K-medium serving as negative control were applied to the cartridges, and eluted with 10 mL water twice and subsequently with 20 mL of 20, 30, 40, 50, 60, 70, 80, 90, and 100% methanol, and finally with 10 mL chloroform/methanol [3:1 (v/v)]. Each eluate fraction except for first 10 mL sample load eluate (salt included) was dried by rotary evaporation and re-dissolved in 5 mL K-medium. Sub-samples of 3.9 mL each as well as the waste from the last 5 mL (195 to 200 mL) were tested as a one-point bioassay. Based on the results of the preliminary tests, the final SPE elution protocol is as follows: after sample (200 mL supernatant) loading the cartridge was washed with 10 mL water, 10 mL 20% methanol, 20 mL 50% methanol and 10 mL 80% methanol.

In order to quantify the lytic potency of two lytic fractions eluted with diverse eluents gained from SPE, 200 mL supernatant was applied to one C-18 cartridge, and the eluates were collected every 20 mL. For the first 5 eluate collections, one-point (3.9 mL) bioassays were performed in duplicates, while a 4-point dilution curve (3.9, 2.0, 1.0, and 0.5 mL) was performed with last five eluate collections in duplicate. The loaded SPE column was eluted as the final protocol.

The 10 mL water and the 10 mL 20% methanol fractions were combined and dried by rotary evaporation, and then were re-dissolved in 20 mL K-medium. To this H_2_O-20% methanol sample, a 5-point dilution series was performed including a negative control (K-medium) to exclude possible salinity effect on *Rhodomonas*. The 10 mL 80% methanol fraction was separated into 2 aliquots. After dried by rotary evaporation, one aliquot was dissolved in 5 mL K-medium, while the other was dissolved in 5 mL leftover of 180–200 mL eluate collection. Bioassay with a 6-point dilution series for both 80% methanol sample and 80% methanol mixed sample as well as supernatant of the same batch in duplicate was performed.

### Effects of SPE fraction storage conditions on lytic activity

Lytic effects of SPE fractions maintained under different storage conditions prior to applying the bioassay were investigated by applying 800 mL supernatant to 4 SPE cartridges (200 mL each) and eluted as described before. All 80% methanol fractions were pooled and split into 4 sub-samples (10 mL each). One sub-sample was stored in 80% methanol (1) in the fridge at 4 °C. All other sub-samples were dried by rotary evaporation. One fraction was stored as dried sample (2), another resuspended in 5 mL water (3) and the last sample (4) re-suspended in 5 mL K-medium. After 2 days, aliquots (1) was rotary evaporated to dryness and then all aliquots were re-suspended in 10 mL K-medium. Immediately, a first bioassay was performed for each sample with a 6-point dilution curve in duplicate. All samples in culture medium were then stored in the fridge at 4 °C for an additional two days; thereafter new bioassays were performed with the same dilution series.

### Reversed phase HPLC

HPLC was performed using an Agilent 1100 series (Agilent Technologies,Waldbronn, Germany) system. The LC-system consisted of a G1379A degasser, a G1311A quaternary pump, a G1229A autosampler, a G1330B autosampler thermostat, a G1316A column thermostat and a G1315B Diode-array detector (DAD). Chromatographic conditions were as follows: mobile phase A: water; mobile phase B: methanol. The flow rate was 0.2 mL/min with the following gradient: 0 until 15 min, 5% to 100% B; isocratic 100% B until 27 min; 27 until 28 min, 100% to 5% B; isocratic 5% B until 40 min (= total run time). The autosampler temperature was set at 25 °C and the injection volume was 100 μL. One liter supernatant of both Alex2 and Alex5 cultures was cleaned up by C18 SPE (500 mg/6mL, Sigma-Aldrich, Deisenhofen, Germany). The 80% methanol fraction was collected, dried by rotary evaporation, and finally dissolved in 1 mL water. Before injection, the sample was spin-filtered (Eppendorf 5415R, 30 sec, 15,000 × g, room temperature) by filter unit insert (0.45 μm, Durapore, Millipore). The separation of analytes was performed on a 50 × 2.0 mm i.d., 3 μm, Hypersil BOS C8 reversed-phase column (Phenomenex, Aschaffenburg, Germany) equipped with a Phenomenex SecuriGuard pre-column. Based on preliminary result, eluate was collected very 1 min from 15 to 20 min, and lytic activity was detected in each fraction. Accordingly, the target range was detected over the wave length range 190–400 nm.

### Dry mass equivalent

For the determination the dry mass equivalent of lytic activity, bioassays with dilution series were performed with supernatant, the lytic SPE fraction and the lytic HPLC fraction (details see above experiments). Based on the EC_50_ value, the corresponding volumes of the treatments were calculated. These respective treatment volumes were dried in a N_2_ stream and dry residues were weighed.

## 5. Conclusions

Analysis of physico/chemical characteristics of extracellular lytic compounds produced by *A. tamarense* indicate that these allelochemicals behave like amphipathic compounds. Although temporal stability is high, a large molecular- or aggregate-size and high adsorption caused massive loss of activity during various purification steps. Nevertheless, although the exact chemical nature of lytic compounds produced by *Alexandrium* spp. is still open, we now have the knowledge and tools to handle and prepare larger amounts of samples for further purification through high performance liquid chromatography (HPLC) needed for structural elucidation by mass spectrometric techniques (MALDI-TOF or LC-MS/MS) and nuclear magnetic resonance (NMR) spectroscopy.

## Figures and Tables

**Table 1.** EC_50_ (sample %) and 95% confidence intervals for SPE lytic fraction dissolved and stored in four different ways.

**Treatment**	**EC_50_ (%) pre-treatment**	**EC_50_ (%) after treatment**
80% methanol	3.4 (2.8–4.2)	2.8 (2.7–2.9)
Water	22.2 (19.4–25.4)	5.0 (4.3–5.8)
K-medium	2.1 (1.9–2.3)	2.3 (1.9–2.8)
Dryness	4.1 (3.5–4.7)	2.6 (2.6–2.7)
